# The complete chloroplast genome of *Chimonobambusa hejiangensis* (Poaceae: *Arundinarieae*) and phylogenetic analysis

**DOI:** 10.1080/23802359.2021.1886007

**Published:** 2021-04-01

**Authors:** Yanjiang Liu, Ting Su, MaiMai Peng, Jiaxue Li, Xiao Zhu, Guangqian Gou, Zhaoxia Dai

**Affiliations:** a Key laboratory of Plant Resource Conservation and Germplasm Innovation in Mountainous Region (Ministry of Education), Collaborative Innovation Center for Mountain Ecology & Agro-Bioengineering (CICMEAB), Institute of Agro-bioengineering/College of Life Sciences, Guizhou University, Guiyang, Guizhou Province, China; b Bamboo Research Institute, Guizhou University, Guiyang, China; c College of Forestry, Guizhou University, Guiyang, China

**Keywords:** *Chimonobambusa hejiangensis*, chloroplast genome, Poaceae, phylogenetic relationship

## Abstract

*Chimonobambusa hejiangensis* is a kind of bamboo that has excellent edible and economic value, which is endemic to southwest China. The study used next-generation sequencing to obtain the complete chloroplast (cp) genome sequence of *C. hejiangensis*. The cp genome of *C. hejiangensis* has a total length of 138,908 bp, and consisted of an 82,495-bp large single-copy region, an 12,743-bp small single-copy region, and two 21,835-bp IR regions. In total, 112 unique genes were found in the cp genome, including 77 protein coding, 31 tRNA, and 4 rRNA genes. Phylogenetic analysis indicated that *C.hejiangensis* and *C. tumidissinoda* are sister species within the *Arundinarieae* genus, where *Chimonocalamus* and *Ampelocalamus* are more closely related to them.

*Chimonobambusa hejiangensis* C. D. Chu & C. S. Chao is mainly distributed in southern China at the junction of Guizhou and Sichuan provinces. It is suitable for growing in acidic and neutral soils with an optimal altitude of 180–1300 m (Liu et al. 2012). Previous research has shown that bamboo shoots from *C. hejiangensis* excel in autumn, when they are not only delicious but also contain various trace elements such as iron, sun, and zinc, as well as being rich in other nutrients (Gou et al. 2010).

Chloroplasts are organelles that take part in photosynthesis and known as the ‘nutrient manufacturing workshop’ and ‘energy conversion station’ in plants. From a structural point of view, the two inverted repeats (IRs) of the chloroplast genome are separated by a large single-copy region (LSC) and a small single copy region (SSC), thus forming a typical quadripartite structure. Compared with two other genetic materials, namely, nuclear genome and linear chloroplast genome, the chloroplast genome has characteristics of strong conservatism, easy access to sequences, and moderate rate of evolution, providing sufficient resources for plant systematics (Wu et al. 2009). Related research from morphological identification to the molecular perspective is of great significance in understanding the classification and evolutionary relationships among bamboo species (Zhang et al. 2011; Liu et al. 2018). In recent years, with the emergence of next-generation sequencing (NGS) technology, chloroplast genome sequences have been progressively analyzed, contributing insight into the phylogenetic traits of plants, transgenes, and plant identification (Ceccherini et al. 2003; Wambugu et al. 2015; Xue et al. 2019).

The research materials were collected in Bao yuan Township, Guizhou Province (28°20′14″N and 105°38′32″E). The voucher specimens of *C. hej*iangensis were deposited at the Natural Museum of Guizhou University (accession number: GACP). The chloroplast DNA was extracted using a TIANGEN DNA extraction kit (TIANGEN BIOTECH CO., Beijing China) and sequenced based on the Illumina Hiseq 2500 platform (San Diego, CA). Approximately 3GB of paired-end (150 bp) raw short sequence data were generated. The GetOrganelle (Jin et al. 2020) was used to *de novo* assemble the complete cp genome. PGA (https://github.com/quxiaojian/PGA) annotated the chloroplast genes of *C. hejiangensis* with *C. tumidissinoda* as the reference followed by manual correction using the software Generous 10.0.5 (Kearse et al. 2012), with uploading to NCBI after the sequence was confirmed to be correct (GenBank MT884004).

The chloroplast genome of *C. hejiangensis* with a length of 138,908 bp, and consisted of an 82,495-bp LSC region, a 12,743-bp SSC region, and two 21,835-bp IR regions, respectively, comprising the typical quadripartite structure of terrestrial plants. The total GC content found for the *C. hejiangensis* cp genome was 38.93%, and the LSC region (37%) and SSC region (33.2%) have much lower values than that in the IR region (44.22%). The cp genome of *C. hejiangensis* was annotated with 112 genes, including 77 protein coding, 31 tRNA, and four rRNA genes. Thirteen genes contain one intron (*atpF, ndhA, ndhB, petB, petD, rpl2, rpl16, rps16, trnA-UGC, trnI-GAU, trnK-UUU, trnL-UAA, trnV-UAC*) and only gene *cyf3* includes two introns, while the intron of the gene *clpP* was found to be deleted.

Phylogenetic analysis of ten complete cp genome sequences were selected to construct the phylogenetic tree using RAxML v.8.2.8 (Stamatakis 2014), and the bootstrap replicates parameter was set to 1,000. O*ryza nivara* was selected as an outgroup. The maximum likelihood (ML) tree showed that the genus *Bambusa* constitutes an isolated evolutionary branch, becoming a monophyletic group, a conclusion that is consistent with previous report (Zhang et al. 2019). Also, the reconstructed phylogeny revealed that *C. hejiangensis* is sister to *C. tumidissinoda* with strong support (100%) within the *Chimonobambusa* genus (Figure 1). This cp genome can provide a theoretical basis for the further study of the Bambusoideae.

**Figure 1.**
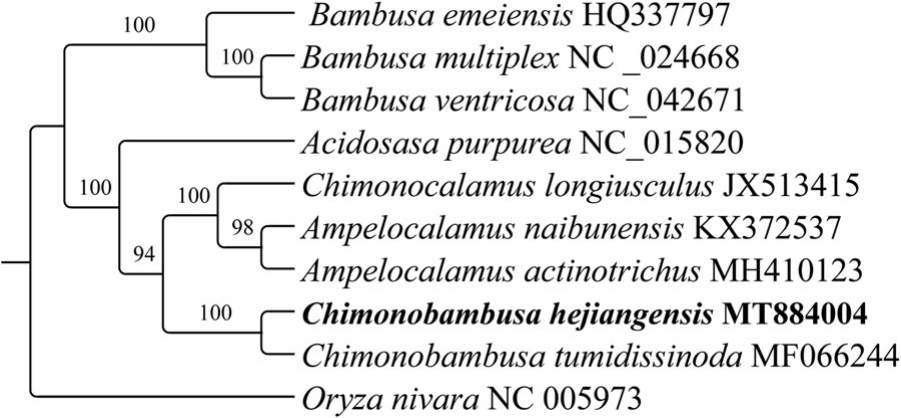
Maximum likelihood phylogenetic tree based on the RAxML software considering 10 Poaceae species and outgroups from *Oryza nivara* (Oryzoideae).

## Data Availability

The genome sequence data that support the findings of this study are openly available in GenBank of NCBI at (https://www.ncbi.nlm.nih.gov/nuccore/MT884004.1/) under the accession MT884004. The associated BioProject, SRA, and Bio-Sample number are PRJNA689348, SRR13346655 and SAMN17207071 respectively.
